# Monitoring physical match performance relative to peak locomotor demands: implications for training professional soccer players

**DOI:** 10.5114/biolsport.2023.116450

**Published:** 2022-07-21

**Authors:** José M. Oliva-Lozano, Andrea Riboli, Víctor Fortes, José M. Muyor

**Affiliations:** 1 Health Research Centre, University of Almería, Almería, Spain; 2 Department of Biomedical Sciences for Health, Università degli Studi di Milano, Milan, Italy; 3 Unión Deportiva Almería, Almería, Spain; 4 Laboratory of Kinesiology, Biomechanics and Ergonomics (KIBIOMER Lab). Research Central Services, University of Almería, Spain

**Keywords:** External Load, Training Load, Game Analysis, Team Sport, Worst Case Scenario

## Abstract

This study aimed to analyse physical performance relative to peak locomotor demands of match play. Data were collected during 13 professional soccer matches. Initially, the 1-minute peak values were registered in each match, including the percentage of the total distance (TD), high-speed running distance (HSRD), sprinting distance (SPD), and high-metabolic load distance (HMLD), and a total of high-intensity accelerations and decelerations (Acc+Dec). Secondly, the time (measured in minutes) spent at different percentage ranges for the 1-minute peak values registered in each match was calculated. Thirdly, the physical performance required in the different percentage ranges for the 1-minute peak values were obtained. Finally, the time and physical performance required above the 90-minute average demands were calculated. The 90-minute average for all playing positions represented ~53% of the total distance (TD), ~23.4% of high-metabolic load distance (HMLD), ~16% of high-speed running distance (HSRD), ~11% of the total of high-intensity accelerations and decelerations (Acc+Dec), and ~6% of sprinting distance (SPD) for the 1-minute peak values. Likewise, statistically significant differences (p < 0.05) in the physical performance and time spent between specific percentage ranges for the 1-minute peak locomotor demands were also noted. In addition, all the variables reported that the physical performance required for above 90-minute average demands were significantly greater (p < 0.05) than the 90-minute average demands. Therefore, these findings may guide the prescription of training intensity by considering the physical performance relative to the peak locomotor demands of match play.

## INTRODUCTION

The application of electronic performance and tracking systems to soccer has increased our understanding of the physical demands of the game [1]. Soccer is characterized by intermittent activity and high-intensity actions, which have increased significantly over the past several years [2], are interspersed with periods of lower intensity [3, 4]. Tracking systems provide objective data about players’ performance, and thus, tracking performance has become a common tool used by strength and conditioning coaches [5].

The management of the data can guide coaches in their decisionmaking process regarding training schedules [6]. Several studies have concluded that professional soccer players should be trained based on the physical demands of match play to prevent injury and ensure successful match performance [3, 7, 8]. For example, a previous study suggested that if the players covered 115 m/min during the match (with an average of 29 m/min at high-speed running), they ought to be trained for similar demands [3]. Hence, coaches may design their training drills (e.g., modifying area per player in small-sided games) to meet such demands [9].

However, recent studies have concluded that the training drills should also consider the periods of peak locomotor demands experienced by the players during match play because 90-minute average demands may be lower than the physical demands experienced in match play [10–12]. Specifically, these periods of peak locomotor demands, which have been interpreted as the most demanding passages of play or worst-case scenarios by previous studies, are duration-specific periods of the match (e.g., 1-minute periods) in which the players are required to perform at very high intensity [4, 11, 13, 14]. For instance, a recent study reported that professional players could cover ~200 m (with ~61 m considered to be high-speed running and ~30 m to be sprinting speed) or perform ~4 high-intensity accelerations or decelerations in one minute [4]. Additionally, the 1-minute periods have been recently described across team formation, ball in play, and ball possession to help practitioners contextualize the maximal technical-tactical match demands during practice [15].

Nevertheless, it should be noted that these peak locomotor demands only occur once throughout a match, and this approach, therefore, does not reflect overall physical demands (especially when it comes to the volume completed at or close to such peak demands) [12, 16]. In this regard, the peak locomotor demands of match play may guide training intensity prescription, although little is known about the distribution of this intensity in professional soccer [12]. For instance, strength and conditioning coaches might wonder which percentage range (for the 1-minute peak locomotor demands) corresponds to the 90-minute average demands and how long the players must train above this intensity [12]. Given that research on the topic is scarce, this study aims to analyse physical performance relative to peak locomotor demands of match play.

## MATERIALS AND METHODS

### Study design

A longitudinal study was conducted in LaLiga123. Data were collected using electronic performance and tracking systems during 13 official 2018–2019 season matches (7 away matches and 6 home matches). These matches were played on a non-congested calendar (i.e., only one match per week) with 4-4-2 as the standard playing formation. This study was conducted in accordance with the principles of the Declaration of Helsinki (1975), and the university’s Bioethics Committee authorized the data collection.

### Participants

Seventeen professional soccer players (26.79 ± 3.72 years old; body mass index, 23.30 ± 0.21) participated in the study. The players were classified according to the following playing positions: fullbacks (FB, *n* = 5), forwards (FW, *n* = 2), central defenders (CD, *n* = 3), wide midfielders (WMF, *n* = 3), and central midfielders (MF, *n* = 4). Only players who played for the full duration of the match were included. Specifically, a total of 109 match observations were registered with an average of 6.4 ± 4.1 matches per player. In addition, goalkeepers were excluded as this playing position is characterized by a different activity profile [17].

### Procedures

WIMU Pro (RealTrack Systems, Almería, Spain) electronic performance and tracking systems were placed in the back pocket of players’ vests (Rasán, Valencia, Spain). These tracking systems are valid and reliable instruments for tracking physical performance in soccer [18] and have been certified by the FIFA Quality Program to collect physical performance variables based on position and speed accuracy [19]. Each device was turned on 30 minutes before each match and calibrated on a Smart Station (RealTrack Systems, Almería, Spain) following the manufacturer’s instructions [20]. Furthermore, each player wore the same device in every match to reduce inter-unit error [4].

Once the match had concluded, the data were transferred to SPro (RealTrack Systems, Almería, Spain). Match data were split into two halves of match play (first and second half), and then, these halves were divided into 1-minute periods. The Intervals Pro performance report (RealTrack Systems, Almería, Spain) was then downloaded. Also, the 1-minute peak locomotor demands were obtained through the worst-case scenario report (RealTrack Systems, Almería, Spain) based on rolling averages techniques [21]. Specifically, the following physical performance variables were analysed: total distance (TD) covered, high-speed running distance (HSRD, above 19.8 km/h), sprinting distance (SPD, above 25.2 km/h), high-metabolic load distance (HMLD, above 25.5 W/kg), and the sum of high-intensity accelerations and decelerations (Acc+Dec, above 3 m/s^2^) per minute.

### Statistical analysis

Once the data was exported, the physical performance analysis relative to the peak locomotor demands of match play was conducted following the procedures used in a previous study [12]. Firstly, the percentage of TD, HSRD, SPD, HMLD, and Acc+Dec for the 1-minute peak values registered in each match was calculated. Secondly, the time (in minutes) spent at different percentage ranges for the 1-minute peak values registered in each match (e.g., time spent between 0% and 10% of the TD covered in the 1-minute peak) was calculated. Thirdly, the volume of physical performance required by a percentage range for the 1-minute peak values was obtained (e.g., TD covered per minute between 90% and 100% of the TD covered in the 1-minute peak multiplied by the time spent within the same range). Finally, to represent the total volume of physical performance at intensities above the 90-minute average demands, a sum of the total volume of physical performance required by a range of percentages for the 1-minute peak values above the 90-minute average demands was calculated.

This study used a linear mixed model analysis to compare the effects of playing position by percentage ranges for the 1-minute peak locomotor demands on each variable related to the physical demands [12]. The model considered each variable representing physical performance parameters (i.e., TD, HSRD, SPD, HMLD, and Acc+Dec), the playing position, and the different percentage ranges for the 1-minute peak locomotor demands as independent fixed factors while the players were set as random intercepts. A log-likelihood ratio test was used to analyse the quality of fit of the models. Multiple comparison analysis was determined using Bonferroni’s correction. Cohen’s *d* effect size (ES) with 95% confidence intervals (CI) calculated and categorized them as trivial (< 0.20), small (0.20–0.59), moderate (0.60–1.19), large (1.20–1.99), and very large (≥ 2.00) [22]. The level of statistical significance was set at α < 0.05. The statistical analysis was performed on SPSS (version 26, Armonk, NY, USA).

## RESULTS

**FIG. 1:**
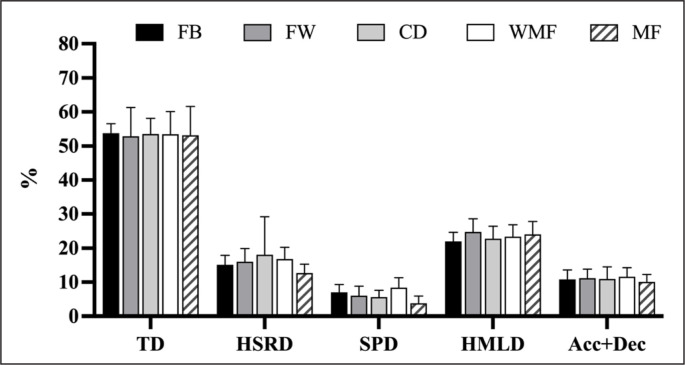
90-minute average demands represented as the percentage of the total distance (TD), high-speed running distance (HSRD), sprinting distance (SPD), high-metabolic load distance (HMLD), and the total number of accelerations and decelerations (Acc+Dec) for the 1-minute peak values registered for all playing positions. Note: FB: fullbacks; FW: forwards; CD: central defenders; WMF: wide midfielders; MF: central midfielders.

Figure 1 shows the 90-minute average demands represented as the percentage of TD (53.3 ± 0.3%), HSRD (15.7 ± 2.0%), SPD (6.2 ± 1.7%), HMLD (23.4 ± 1.1%), and Acc+Dec (10.9 ± 0.5%) for the 1-minute peak values registered in each match for all playing positions. In this regard, substantial differences between variables (*p* < 0.001; ES: 2.07 to 38.4) were observed for all playing positions. However, no significant differences between playing positions (*p* > 0.05) were found.

**FIG. 2:**
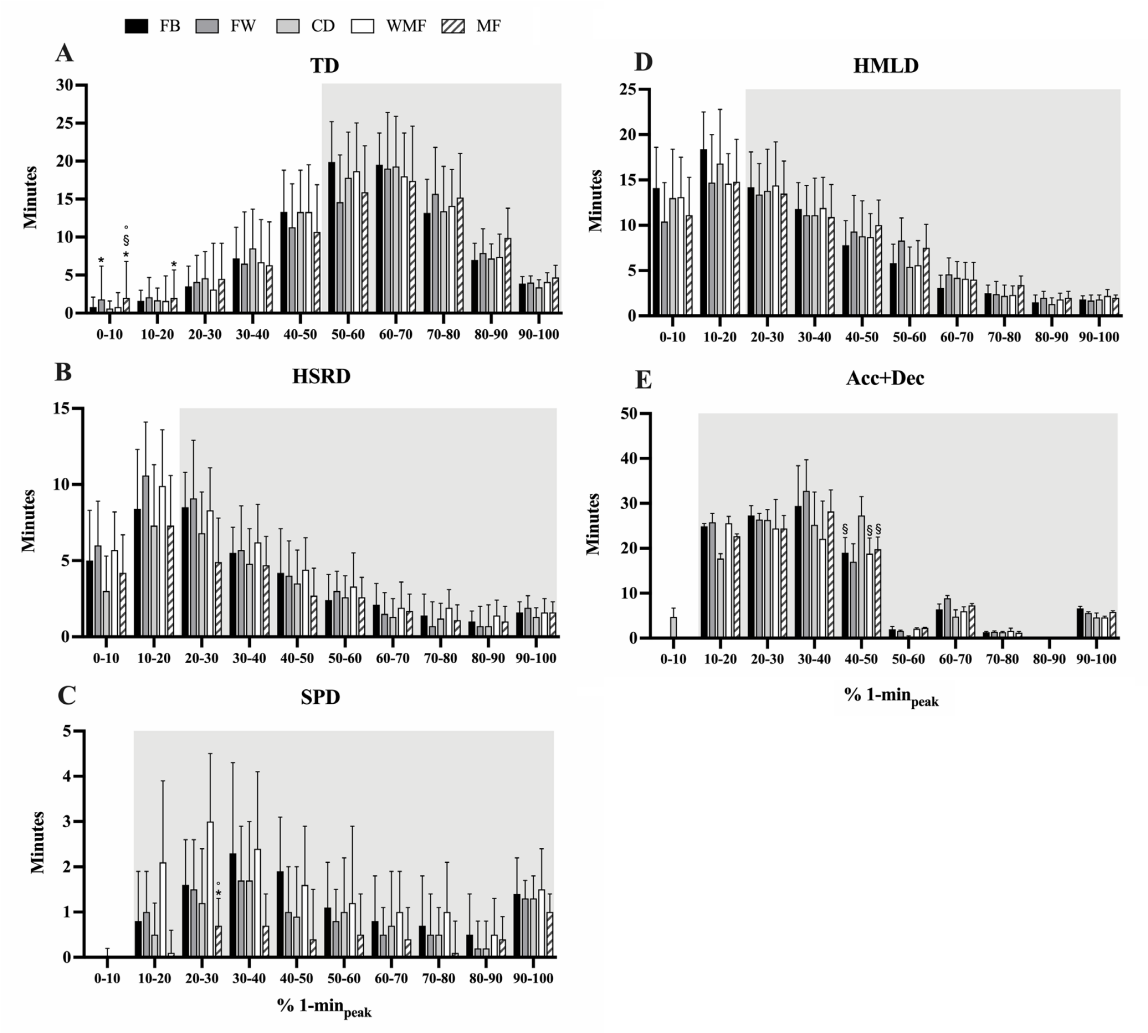
Time (in minutes) spent at different ‘percentage ranges for each playing position’s 1-minute peak locomotor demands. The grey area highlights the time required for above 90-minute average demands. Note: TD: total distance; HSRD: high-speed running distance; SPD: sprinting distance; HMLD: high metabolic load distance; Acc+Dec: a total of high-intensity accelerations and decelerations. FB: fullbacks; FW: forwards; CD: central defenders; WMF: wide midfielders; MF: midfielders. ^*^p < 0.05 *vs* FB; ^§^*p* < 0.05 *vs* CD; °*p* < 0.05 *vs* WMF.

Figure 2 shows the time (in minutes) spent at different percentage ranges for each playing position’s 1-minute peak locomotor demands. There were significant differences in time spent between specific ranges (*p* < 0.001). In addition, although the main effect of playing position on the time spent at different percentage ranges for the 1-minute peak locomotor demands was not significant (*p* > 0.05), some differences between playing positions were observed. In this regard, trivial to small differences between positions (ES: 0.04 to 0.37) in TD and Acc+Dec as well as a moderate difference in forwards compared to midfielders at the 20–30% range for SPD were found (Figure 2).

Specifically, Table 1 shows the volume of physical performance required for each percentage range (e.g., TD covered per minute at each range multiplied by the time spent at the same range) for the 1-minute peak locomotor demands. Significant differences in physical performance were required between specific ranges (*p* < 0.001) for all variables.

**TABLE 1:** The volume of physical performance required for each percentage range (e.g., TD covered per minute at each range × the time spent at the same range) for the 1-minute peak locomotor demands

Ranges	TD (m)	HSRD (m)	SPD (m)	HMLD (m)	Acc+Dec (count)
0–10%	16.5 ± 8.5	30.2 ± 10.7	0.1 ± 0.1	75.1 ± ± 12.5	0.0 ± 0.0
10–20%	77.9 ± 22.7	104.3 ± 26.4	**8.3 ± 7.2**	212.2 ± 28.8	**1.5 ± 1.2**
20–30%	261.9 ± 55.5	**136.4 ± 45.1**	**18.3 ± 11.1**	**290.1 ± 32.9**	**6.2 ± 3.8**
30–40%	572.4 ± 82.6	**126.6 ± 29.5**	**27.5 ± 13.7**	**319.4 ± 45.2**	**26.8 ± 6.2**
40–50%	1192.3 ± 51.9	**109.3 ± 33.3**	**21.7 ± 13.6**	**318.1 ± 41.3**	**22.0 ± 4.4**
50–60%	**1886.7 ± 164.8**	**93.4 ± 24.8**	**19.8 ± 9.1**	**258.3 ± 43.5**	**0.2 ± 0.3**
60–70%	**2252.2 ± 163.2**	**66.3 ± 18.6**	**16.7 ± 7.1**	**187.9 ± 28.1**	**12.9 ± 3.6**
70–80%	**1902.8 ± 177.7**	**58.4 ± 25.1**	**16.9 ± 11.6**	**130.2 ± 22.7**	**1.4 ± 1.1**
80–90%	**1100.7 ± 143.2**	**47.9 ± 19.9**	**11.5 ± 6.8**	**103.6 ± 23.3**	**0.0 ± 0.0**
90–100%	**654.0 ± 100.0**	**94.0 ± 20.5**	**48.3 ± 12.9**	**127.3 ± 24.9**	**12.4 ± 1.4**

Note: TD = total distance; HSRD = high-speed running distance; HMLD = high-metabolic load distance; Acc+Dec = total of high-intensity accelerations and decelerations. Bold text highlights the physical performance required above the average percentage corresponding to the 90-minute demands.

The results reported that the physical performance required above 90-minute average demands was significantly greater (*p* < 0.001; ES: 2.35 to 13.33) than the 90-minute average demands in TD (125.9 ± 6.2 m/min vs 107.2 ± 9.3 m/min), HSRD (29.3 ± 3.8 m/min vs 9.1 ± 2.9 m/min), SPD (20.2 ± 1.5 m/min vs 2.0 ± 1.2 m/ min), HMLD (34.4 ± 3.68 m/min vs 21.4 ± 3.9 m/min), and Acc+Dec (1.3 ± 0.1 actions/min vs 0.9 ± 0.2 actions/min). In this regard, Table 2 shows the total volume of physical performance at intensities above the 90-minute average demands. Specifically, the 78.7 ± 1.8%, 84.5 ± 1.5%, 99.9 ± 0.2%, 85.8 ± 1.9% and 100.0 ± 0.0% of total volume for TD, HSRD, SPD, HMLD and Acc+Dec, respectively, was performed above the 90-minute average demands.

**TABLE 2:** The total volume of physical performance at intensities above 90-minute average demands.

	TD	HSRD	SPD	HMLD	Acc+Dec
**Minutes**	62.2 ± 1.1	24.9 ± 3.6	9.3 ± 3.7	50.9 ± 2.4	64.5 ± 4.0
**Meters**	7834.8 ± 454.4	739.6 ± 194.6	190.0 ± 89.2	1748.5 ± 213.6	n/a
**Count**	n/a	n/a	n/a	n/a	83.5 ± 8.3

Note: TD = total distance; HSRD = high-speed running distance; HMLD = high-metabolic load distance; Acc+Dec = total of high-intensity accelerations and decelerations; n/a = not applicable.

**FIG. 3:**
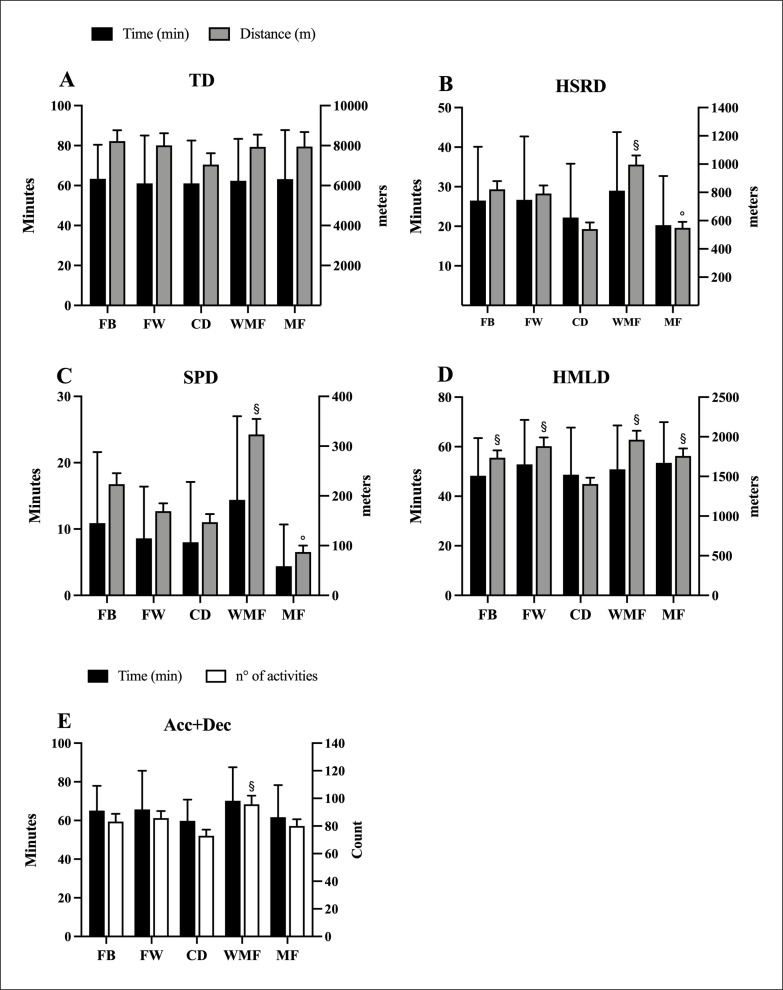
Time and physical performance required above 90-minute average demands by playing position. Note: TD: total distance; HSRD: high-speed running distance; SPD: sprinting distance; HMLD: high metabolic load distance; Acc+Dec: a total of high-intensity accelerations and decelerations. FB: fullbacks; FW: forwards; CD: central defenders; WMF: wide midfielders; MF: midfielders. ^§^*p* < 0.05 *vs* CD; °*p* < 0.05 *vs* WMF.

Figure 3 summarizes the time and physical performance required above the 90-minute average demands by playing position. No significant differences (*p* > 0.05) between the playing positions were found for the time spent above the 90-minute average demands. However, some differences were found between positions (*p* < 0.05) for HSRD, SPD, HMLD, and Acc+Dec required above the 90-minute average demands (*p* < 0.05; ES: -1.83 to -0.25). Specifically, CD showed significantly lower HSRD (Figure 3B), SPD (Figure 3C), and Acc+Dec (Figure 3E) than WMF. Also, CD covered the lowest HMLD compared to the rest of the playing positions (Figure 3D).

## DISCUSSION

This study analysed physical performance relative to peak locomotor demands of match play. One of the main findings of this study was that 90-minute average demands represented ~53% of TD, ~23.4% of HMLD, ~16% of HSRD, ~11% of Acc+Dec, and ~6% of SPD for the 1-minute peak locomotor demands for all playing positions. Another novel finding was the differences in the volume of physical performance and time spent between specific percentage ranges for the 1-minute peak locomotor demands. In addition, the physical performance required for above 90-minute average demands was significantly greater than the 90-minute average demands in all variables, especially for the most demanding locomotor actions (i.e., SPD and Acc+Dec).

The fact that the 90-minute average represented ~53% of TD, ~23.4% of HMLD, ~16% of HSRD, ~11% of Acc+Dec, and ~6% of SPD for the 1-minute peak values implies that strength and conditioning coaches should not limit themselves to considering average demands. These findings are in line with previous studies that suggest that 90-minute average demands might under prepare players for higher intensity periods of match play [10–12, 23]. This becomes especially important for variables closely related to high-intensity actions (e.g., HMLD, HSRD, SPD, and Acc+Dec) since the 90-minute average demands represented very low values in the 1-minute peak values. In this regard, a recent study reached a similar conclusion, with the 90-minute average representing ~60% of TD and only ~9% of SPD for the 1-minute peak values, regardless of the playing position [12]. Moreover, another recent study, which focused on the analysis of the physical demands of transitions comparing them to 90-minute match demands, observed that the transitions exceeded the 90-minute average in all players and exposed players to maximal physical outputs [23].

This study also found differences in the volume of physical performance between specific percentage ranges for the 1-minute peak locomotor demands. For instance, players spent more time within the 60–70% range of peak locomotor demands compared to other ranges and covered ~2252 m within this range. This is consistent with a recent study that analysed a larger sample (n = 148). Said study showed players spent more time within the 60–70% range of peak locomotor demands in TD than in other ranges and covered ~2580 m within this range [12]. Similar to the present findings, the same study [12] found that players covered more SPD at 90–100% of peak locomotor demands. Consequently, since the distribution of this intensity in professional soccer matches was analysed [12], these findings may guide training intensity prescription.

Furthermore, this study observed that the physical performance required above 90-minute average demands was significantly greater than the 90-minute average demands in all variables. A high percentage of the total distance covered across different metrics during official matches was at a higher intensity than the 90-minute average demands. In this instance, ~79% to ~85% of the total whole match distance for TD, HSRD, and HMLD was at a higher intensity than the 90-minute average match demands. Interestingly, for the most demanding metrics (i.e., SPD and Acc+Dec), the whole (i.e., ~100%) locomotor load was covered at a higher intensity than the 90-minute average match demands. Previous findings have highlighted similar results, with ~95% of the SPD covered above the 90-minute average demands in Italian Serie A soccer players [12]. Thus, coaches and sports scientists should consider the physical performance over the 90-minute average match demand for more contextualized soccer-specific training prescriptions, especially for high-intensity activities. In this regard, having a better understanding of when and how soccer players perform high-intensity activities would be interesting too from a practical perspective [24, 25].

This study presents some limitations which need to be acknowledged. For example, only five key load indicators in professional soccer were used. Some of these variables, which included high-speed and high-intensity acceleration actions, were based on absolute thresholds (e.g., 25.2 km/h for SPD). However, the individualization of these variables (e.g., relative to a player’s maximum speed) may be considered for future studies [26]. In addition, although this type of analysis is recommended to be used by each soccer team and similar results were observed in previous research [12], our sample was limited to one professional soccer team. Lastly, practitioners should acknowledge that the maximal individual locomotor, cardiovascular and metabolic capacity could exceed maximal individual match requirements. Therefore, while match-derived training prescription is critical for high-performance development, specific exercises based on individual cardiovascular and metabolic capacity at submaximal [27] and maximal intensities [28, 29] should be considered.

## CONCLUSIONS

These findings may guide the prescription of training intensity. The total volume of physical performance required for above 90-minute average demands could be employed to design the whole training session load. If the 90-minute average represented ~53% of TD covered for the 1-minute peak locomotor demands, this implies that coaches should not only focus on match averages as reference values. The time and physical performance required above 90-minute average demands should be used as a reference for designing the whole training session. In addition, the volume required at different ranges of percentages for the 1-minute peak locomotor demands may be helpful for the design of specific drills (e.g., small-, medium- and large-sided games or positional drills). For example, if the HSRD covered by the players is ~50 m in the 1-minute peak locomotor demands and matches also require ~6 minutes above 80% for the 1-minute peak locomotor demands, coaches may design positional games that take into account this time window and intensity (e.g., six repetitions of 1 minute).
